# Designing Maker initiatives for educational inclusion

**DOI:** 10.1007/s10798-022-09754-1

**Published:** 2022-04-22

**Authors:** Simon N. Leonard, Manuela Repetto, JohnPaul Kennedy, Enza Tudini, Samuel Fowler

**Affiliations:** 1 Centre for Change and Complexity in Learning, University of South Australia, Adelaide, Australia; 2 Department of Philosophy and Educational Sciences, University of Turin, Turin, Italy; 3 Centre for Research in Educational and Social Inclusion, University of South Australia, Adelaide, Australia

**Keywords:** The Maker movement, STEM education, Inclusive education, Equity, Student attitude survey

## Abstract

The “Maker” movement is a cultural as well as educational phenomenon that has the potential to offer significant opportunities to students in conditions of social, economic and cultural disadvantage. The research reported in this paper, however, suggests that the simple provision of “Maker Spaces” for such activity is simplistic and not sufficient to realise this potential. The research involved a mixed methods study of a cohort of year 7 students (n = 26) in an Australian school located in a socio-economically disadvantaged outer-metropolitan region. The cohort undertook a range of Maker activities at a new “creativity centre” built at the school. Results indicate that the activities had positive impact on student attitudes towards science, technology, engineering and mathematics (STEM) overall, but that the impact was highly specific across attitudinal constructs. A strong ranging effect was also evident, suggesting that the impact of the experience was highly dependent on students’ initial attitudes. Reflecting on these results, the paper also offers a reference framework that may help keep equity in mind when designing different kinds of Maker experience.

## Introduction

The “Maker” movement is spreading internationally as a cultural and educational movement focused on the innovative use of digital tools. It combines playful and creative approaches with scientific activities inspired by industrial design and engineering. According to one of the proponents of this movement Blikstein (2014), the processes of ideation, design and implementation inherent in this approach are traceable to the key concepts of Papertian constructionism (Papert, 1993) and have their roots in the pedagogical activism of Dewey and Montessori. In this approach learners—as “Makers”—actively and experientially build their knowledge through practical activities that combine the use of manual and digital skills to create physical or digital artifacts that address open problems inspired by everyday life (Tan et al., 2021).

Maker activities are diverse, including processes such as manufacturing, robotics, cooking, fabric creations, circuit making and 3D printing. What connotes them as a *Maker* activity is the combination of physical objects with digital technologies or applications. This can take two main forms—the use of digital technologies to create physical objects, or the creation of objects that incorporate technological elements (Godhe et al., 2019), such as wearable fabrics made with circuits, or the 3D printed bionic hand (Li, 2018). In either approach, artifacts are created and iterated using programming (coding) and manual production, offering experiences of exploration and manipulation of the physical world supported by technology.

While now quite common in industrial contexts, the technologies typically used within Maker movement are not typically within the reach of most young people. The popularity of the Maker movement, however, has seen the widespread development of “Maker Spaces”. Situated within specialist science, technology, engineering and mathematics (STEM) education facilities, and also in non-STEM spaces such as public libraries, these spaces usually provide ad hoc access to the technologies that underpin Maker movement. This provision is usually made with an explicitly democratic or inclusionary ethos. Maker Spaces are intended to give everyone—or at least everyone within the providing educational institution—access to a range of often very expensive technologies. Researchers such as Barton and Tan (2019) have shown how this access can be used to develop culturally relevant, locally grounded Maker projects.

Other research, however, has suggested that Maker spaces—and Maker movement more generally—may actually be increasing the *exclusion* of some segments of the population and further broadening the digital divide (Barton & Tan, 2019; Campos et al., 2019; Hsu et al., 2017; Vossoughi et al., 2016; Yao et al., 2020). This body of research does not run counter to projects such as that reported by Barton and Tan (2019). Rather it points to the need for careful design, and probably also to a need for resourcing. A common issue identified in this research is that, in real practice, the Maker movement tends to rely on established capacities or know-how. This seems to be evident at the level of interest—students with an existing interest and know-how are more likely to self-select into the informal learning offered in the Maker space. Importantly, it is also likely to occur at the level of capacity—Maker educational designs tend to rely on high levels of student self-regulation that is less likely to be found in disadvantaged populations (Evans & Kim, 2013; Flouri et al., 2014; O'Connor et al., 2019).

This paper will contribute to research on equity in the Maker movement in two ways. First, after a theoretical overview, the paper will provide a brief report on findings from a mixed methods study of a cohort of year 7 students in an Australian school located in a socio-economically disadvantaged outer-metropolitan region. The study, aspects of which have been reported in more detail elsewhere (Fowler et al., 2021), considered student engagement in a relatively highly structured Maker program in a new “creativity centre” recently opened at their school. It measured short term changes to various aspects of student attitude as well as changes in certain cognitive capacities such as spatial reasoning pre and post the engagement with the Maker space.

## Theoretical overview

Literature suggests that many approaches to Maker movement are rooted in neoliberal models of education. They emphasise individual empowerment through the development of personal skills (Weinstein et al., 2016). The risk is that learners who take part in Maker activities founded on these approaches only develop the individual dimension and that competition can replace interaction and collaboration between peers—and possibly even displace learning. Schools that promote Maker initiatives frequently resort to local and international competitions to provide the context that may be missing in the actual curriculum. In doing so, they appeal to the most “talented” students’ pursuit of excellence rather than addressing the entire student population (Yao et al., 2020).

This neoliberal trend, which is at the expense of inclusion and educational equity, reflects the dominant narratives of the economic and political sector around strengthening of the workforce in the STEM fields (Weinstein et al., 2016; Zheng, 2019). This trend has made the implementation of Maker practices technocentric and focused on the development of technical skills, dampening interdisciplinarity, collective creativity and opportunities for personal meaning making outside of future STEM employment. The emphasis on technologies and their use, as well as on the development of skills that only enhance the individuality of the students, distracts those proposing these activities from the importance of focusing instead on creating solutions to problems posed by learners or assigned by teachers to address the subject specific needs of the students (Unterfrauner et al., 2020). Moreover, the uncritical and non-pedagogically founded adoption of the Maker approach in several contexts like in the school setting can even exacerbate inequalities and discrimination, depriving children and young people of an opportunity that could be more exploited.

Alternatives are available. Vossoughi et al. (2016), for example, underline the option of redefining Maker learning through pedagogical practices rooted in the stories, needs and experiences of the most disadvantaged students. All learners require involvement in creative, interdisciplinary and relevant experiences appropriate for their needs that give them access to the tools, opportunities and knowledge from which the disadvantaged are often excluded. It is necessary, perhaps, to start from the identities of the students and the communities to which they belong in order to be able to set up the pedagogical planning and the educational practices to enable the creation of Maker experiences achieving higher levels of educational equity. This contextual consideration positions students as having educational, artistic and cultural value whilst utilising their experiences to broaden and reify disciplinary knowledge. Further effort could similarly be made in exploring the scientific nature of these practices, integrating them into activities that supply practical purpose to the use of digital tools.

In another alternative offered by Tan et al. (2018) community ethnography, consisting in studying cultures from the point of view of an insider, provides a pedagogical approach that promotes equitable Maker initiatives. They claim that students should be equipped with ethnographical skills such as designing surveys or conducting interviews, to obtain data meaningful for their community that could inform the development of their own Maker projects. Thus, their Maker projects acquire an added value from a social and cultural point of view and transform the students into innovative community Makers. A similar approach is that of critical ethnography, a justice-oriented method that makes sense of how youth at margins may engage in Maker activities (Greenberg et al, 2020).

Practice theories derived from Bourdieu (1977) could also offer a deeper way to understand what Maker spaces are about and how could be better used in a more equitable and inclusive direction. Practice theories can be used as lenses for examining a social phenomenon like the Maker movement in the educational field. According to the theory of practice architectures (Kemmis et al, 2014), a practice is socially established human activity involving forms of understandings (sayings), modes of action (doings) and ways in which people interact (relatings). These three elements are connected in specific ways within each initiative of Maker education: the interplay between semiotic, physical and social space takes place in the practice architectures available for students, who should perceive Maker practices as authentic and connected with their real world. The Theory of Practice Architectures may be considered one of the reference frameworks giving a theoretical foundation to the study and inspiring the development of a novel theoretical framework suggesting principles for the equitable implementation of Maker initiatives.

## Study’s research approach

We report here on findings from a study within an ongoing educational design research project conducted in partnership with a very large multi-campus Preschool-Year 12 College. We particularly focus on one mixed methods study into Year 7 s (12–13 year olds) attitude change after Maker experience. The project was conducted with the approval of the University’s Human Research Ethics Committee, the board of the College, and the parents of the children who participated.

Situated in an outer-metropolitan location, this College services a mixed demographic that is broadly representative of the wider Australian population. This relatively even socio-economic distribution—there are about 25% of students in each quartile of the Index of Socio-economic Advantage (ICSEA)—is quite unusual in Australian schools where schools more typically have a skewed distribution. This College is not a “rich” school, nor is it a “poor” school. It is a school where rich and poor are present in equal number.

The ongoing project is focussed on developing curriculum and pedagogy for a new facility within the college known as *The Innovation and Creativity School* (TICS) that was to serve as a central place for students from across all campuses to engage in Maker movement programs, as well as other STEM and Arts courses. In its opening year this involved students spending an entire week at TICS in lieu of their regular class schedule. Our project sought through a number of studies to investigate the impact of Maker activities on a range of variables including student attitudes, spatial reasoning, creativity, and self-regulation of learning. In this paper our focus will be on changes found in specific constructs of student attitude with respect to the core STEM subjects of science and mathematics following the week at TICS doing Maker activities.

Notably, the study took place in the early stages of the COVID-19 pandemic and was suspended for some time due to related school closures. The overall and ongoing impact of the pandemic on the students involved is unclear. However, using data from this and other projects, we have shown elsewhere (Fowler et al., 2021) that the disruptions of the first few months of the pandemic did not appear to impact significantly upon student attitudes. However, the pandemic did disrupt our data collection and we report here only on the one class of 18 female and 16 male Year 7 students with whom we were able to collect data in the week prior to and the week following the time at TICS. This class engaged in a week-long Maker experience that was quite heavily structured by teachers with specialist knowledge in digital technologies. The Maker experiences included five themed activities: GPS in sport, webpage design, creative digital storytelling, 3D modelling, and robotic engineering.

The focus on a single class does limit the claims we can make with the data reported below. Furthermore, the selection of a clustering solution for longitudinal data is by necessity a heuristic process. However, multiple clustering indices were calculated for each solution and provide sufficient confidence to substantiate the key finding that the impacts of the Maker experiences on students’ attitudes are “ranged” or stratified—essentially, they tend to fit to high, mid and low groupings for each construct. We would be less confident in claiming that the specific findings we report here are generalisable. We are not, for instance, suggesting that the high groups we see in this study are representative of similar groups everywhere, and we note the need for further research to develop such generalisable understandings. We are confident though, that the data here provides a compelling case for the need to develop more nuanced understandings of the impact of Maker learning.

The data we report here is drawn from the School Attitude Survey (SAS) instrument (Kennedy et al., 2016). The SAS measures students’ self-reported attitudes towards all their curriculum subjects (mathematics, science, history and so on) across a range of attitude constructs including subject specific anxiety, opportunities for creativity, perceived difficulty, subject enjoyability, intention to continue study at higher levels, subject relevance, self-efficacy, career usefulness, and personal usefulness.

The SAS uses a digital tool in which students move on-screen “sliders” to rate their relative agreement with specific statements for each of their school subjects. This effectively results in an analogue scale from − 50 to + 50 for each attitudinal factor for each subject. By calculating a mean rating for each attitudinal construct across all subjects, a student’s composite attitude rating (CAR) is generated. This CAR can then be subtracted from that student’s ratings for each subject in turn to generate a subject attitude rating (SAR). The SAR is therefore a student’s rating for a particular subject relative to their attitude rating for school in general. This approach to measuring the relative contribution of different subjects and attitudes to a student’s overall attitude profile is similar to the determination of a *Z-score* and allows for individual subjects or the separate attitudinal factors to be compared between groups of students without the need for a universally agreed upon concrete definition of *neutral* attitude (see Kennedy et al., 2016 for detailed explanation of the statistical method).

In this paper we will report specifically on the attitude constructs of self-efficacy, usefulness and enjoyability. These three attitude constructs were of particular interest in this project as they are correlated highly with measurements of creativity made using the SAS (Fowler et al., 2021) and with separate student creativity measurements made prior to the TICS experience (see Kaufman et al., 2021; Tubb et al., 2020 for some of the instruments used). Creativity had been a key concern in a project centred at the newly constructed TICS, and a factor linking self-efficacy, usefulness and enjoyability in the SAS had been found to be strongly associated with student creativity across all school subjects.

Each of the SARs for each student who completed the pre and post SAS measurement for science (n = 26) and mathematics (n = 27) were plotted as a spaghetti plot using R 4.0.5 (R Core Team, 2021). A linear trajectory for each student was then calculated and a k-means style clustering technique used to determine clustering solutions for two to six clusters using the *kml* package in R (Genolini & Falissard, 2010; Genolini et al., 2015). By default, the kml algorithm calculates five standardised clustering indices namely Calinski and Harabasz criteria (original, Kryszczuk and Genolini variants), Ray and Turi criterion, and Davies and Bouldin criterion (see Genolini et al., 2015 for full details). For each attitudinal construct, the clustering solution that maximised the majority of these indices was adopted as being the most likely solution.

**Fig. 1:**
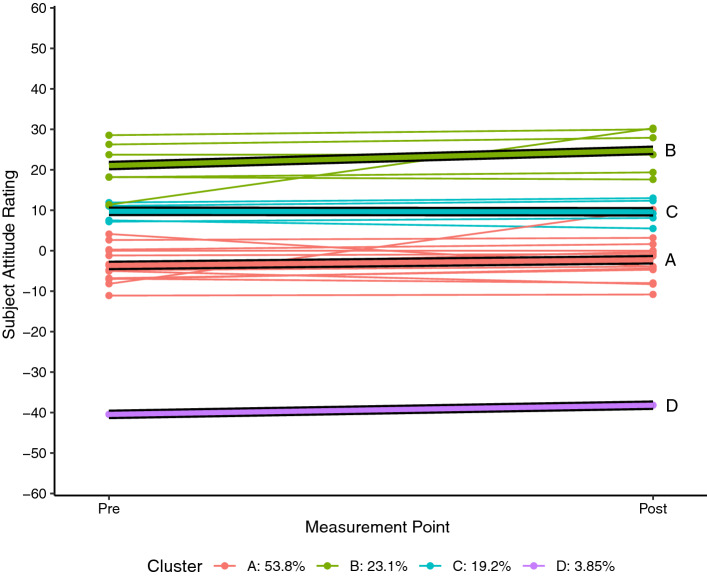
Changes in science Self efficacy

**Fig. 2:**
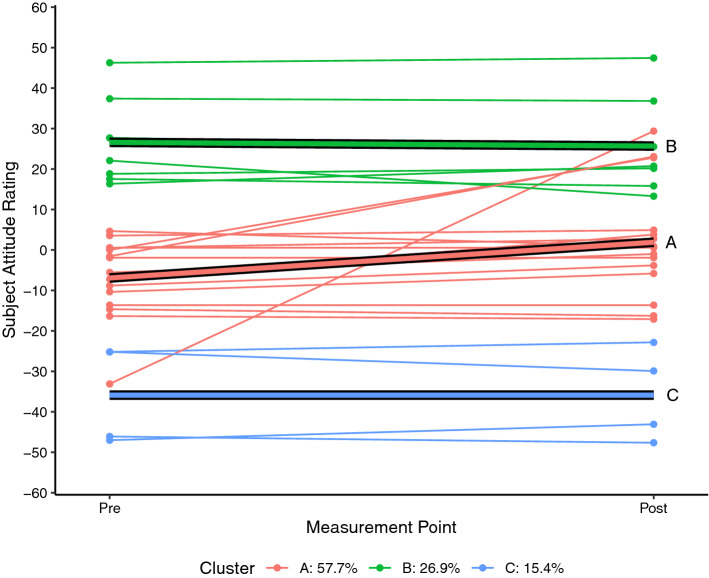
Changes in science enjoyability

**Fig. 3:**
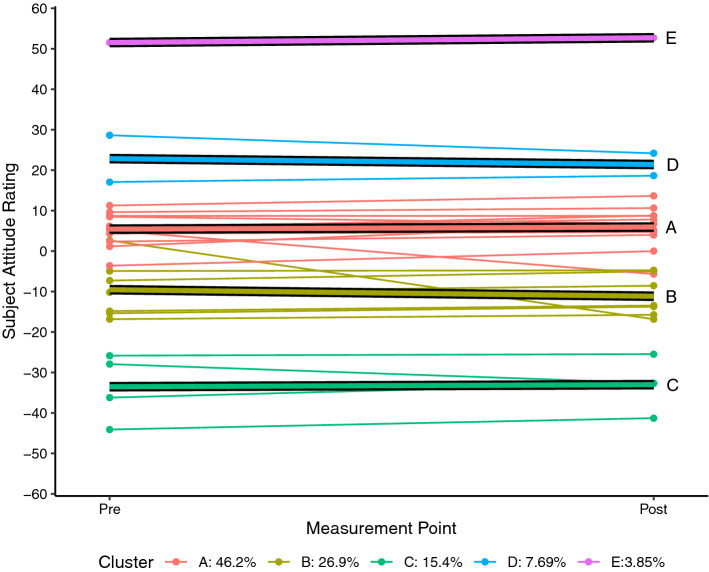
Changes in perceptions of the relevance of Science

**Fig. 4:**
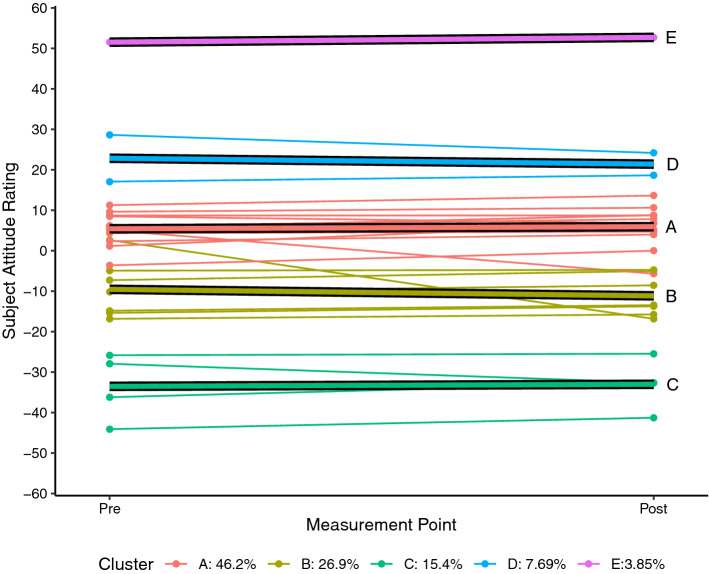
Changes in mathematics Self efficacy

**Fig. 5:**
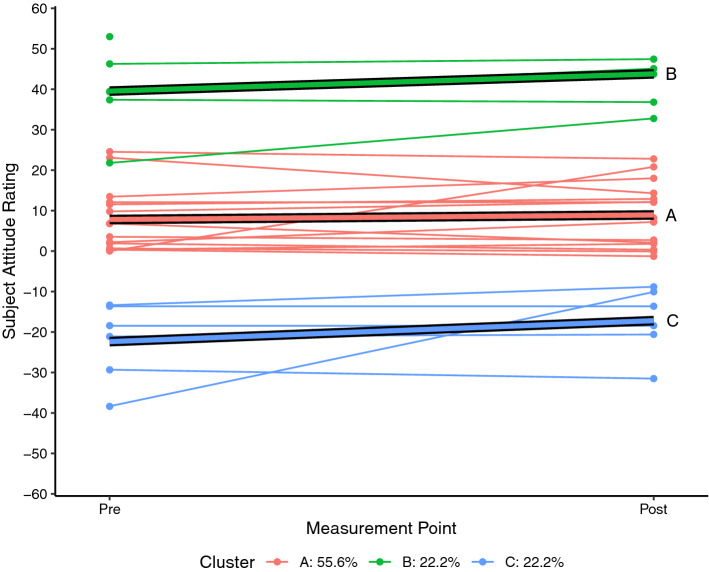
Changes in mathematics enjoyability

**Fig. 6:**
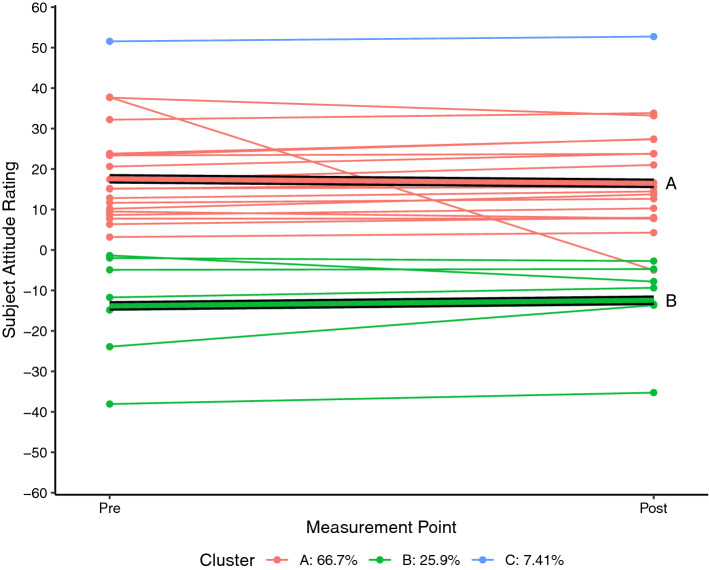
Changes in perceptions of the relevance of Mathematics

Each of the figures that follow show the students’ trajectories in the specific attitude construct with respect to either science (Figs. 1, 2, 3) or mathematics (Figs. 4, 5, 6) between the pre visit measurement (time 1) and the post visit measurement (time 2). The figures show a trajectory for each individual, coloured by cluster, overlaid with a mean trajectory for the cluster as a whole. Each mean trajectory can be described in the following form:

$$SAR={\beta }_{0}+{\beta }_{1}t$$

where β_0_ is the mean SAR for the cluster prior to the visit to the Maker space, and β_1_ is the change in mean SAR post visit.

The legend at the base of each figure provides the proportion of the students assigned by the kml algorithm to each cluster. Clusters are identified by a single letter assigned in order of decreasing proportion of students. We will describe the differences in the trajectories for different groups of students for each of the three attitude constructs across the two school subjects below. As noted, however, the key point we wish to highlight in this paper does not lie in the detail but in simply the fact that the trajectories are not uniform. We see attitudes change not only to different degrees, but in different directions between different groups within the cohort.

## Findings from a Maker project

In this study, the grouped differences in attitude trajectory were highly evident with respect to self-efficacy and enjoyability in science. In Fig. 1 we see four groupings emerge with respect to self-efficacy in science compared to other subjects, although the fourth “group” is a single student whose self-report on self-efficacy was exceptionally low (β_0_ =  − 40, β_1_ =  + 2.3). Of the other groups, we see a clear increase in self-efficacy among the group already rating themselves as highly able (cluster B, β_0_ = 21.0, β_1_ =  + 3.8), while the remaining students with neutral (cluster A) or moderately-positive (cluster C) initial self-efficacy in science report little change (β_1A_ =  + 1.4, β_1C_ =  − 0.1) after their time at TICS.

These differences are even more pronounced with respect to the enjoyability of science, although the pattern is different. In Fig. 2, the group reporting high levels of enjoyment of science (cluster B) actually report a small decline in their enjoyment (β_1_ =  − 0.9). There is a moderate positive correlation between the initial SARs of these two constructs (r(59) = 0.47, p < 0.001), so we are largely seeing the same group of students being clustered into the “initially high rating” group for both enjoyability and self-efficacy, but those students are reporting trajectories with slopes in different directions with respect to the two constructs. The reasons for this are not clear. Perhaps the week of Maker experiences was useful in confirming efficacy but actually not that enjoyable. Or perhaps the Maker experience was highly enjoyable and in their post-experience survey the students are reflecting negatively on their return to regular classes. Regardless of the cause, the important thing for now is that for both self-efficacy and enjoyability, the high group showed a markedly different set of trajectories to the other groups. In Fig. 2 it can be seen that there is a strongly upward trajectory for the neutral attitude group (cluster A, β_0_ =  − 6.9, β_1_ =  + 8.8) while the negative attitude group (cluster C) reported no change in attitude (β_1_ = 0.002). We also find that of the students clustered in the neutral self-efficacy attitude group (Fig. 1 cluster A), 71% of these are also assigned to the neutral attitude group with regards to the enjoyability of science (Fig. 2 cluster A).

When interpreting the results for the construct of *relevance* it is important to recall that the SAS instrument reports the subject or SAR figures as relative to the CAR—the composite rating for all of schooling. As seen in Fig. 3 the majority of students report a SAR close to zero; clusters A and B together account for 73% of students. This indicates that they essentially see science as no more—but also no less—relevant than the rest of their schooling. It is also evident from Fig. 3 that the trajectories of the majority of students are relatively flat; that is there is very little change in students’ reported attitudes towards the relevance of science. Clusters A and C both show minimal positive change (β_1_ =  + 0.5 for both clusters) while clusters B and D show a small negative change (β_1B_ =  − 1.6, β_1D_ =  − 1.5). Cluster E represents a single student who reported that science was far more relevant to them than any of their other subjects.

Taking the above findings all together, we can say that for the majority of students science is as relevant to them as their other subjects and that following the visit to TICS these initially neutral students drifted very slightly away from the mean. That is, those students who rated science as slightly positive became a little more positive, while those who rated it slightly negative became more negative. Importantly however, the group differences we have highlighted with respect to self-efficacy and enjoyability are not evident for this construct. Rather, we see that the Maker experience had virtually no impact on student perceptions of the relevance of science irrespective of the students’ initial SAR. It is also worth noting here that the instrument positions the relevance construct in the present. This construct investigates perception of personal relevance to the student’s current life and interests while perceptions of relevance and usefulness to their future career are explored in another item that is not reported in this paper.

Figure 4 reports self-efficacy in mathematics. The patterns are not as pronounced as were seen in science self-efficacy in Fig. 1, but they are similar. Two clusters of student trajectories emerge from the analysis. The group with high self-efficacy (cluster B, β_0_ = 24.2) show a moderate improvement in self-efficacy (β_1_ =  + 2.7) over the course of the TICS visit. The remainder of the class group (cluster A) reported neutral levels of self-efficacy (β_0_ =  − 0.2)—their self-efficacy in mathematics is similar to their self-efficacy for schooling in general—and while their self-efficacy does increase (β_1_ =  + 1.3) following the Maker experience this increase is less marked than the high self-efficacy group.

Figure 5 shows a somewhat similar pattern to Fig. 4. The group initially reporting high levels of enjoyability (cluster B, β_0_ = 39.6) in mathematics report a clear increase in enjoyability (β_1_ =  + 4.3) following the visit to TICS, while the moderate group show only a small change (cluster A, β_0_ = 7.8, β_1_ =  + 1.1). For the enjoyability construct there is also a third group (cluster C) who reported initially that they found mathematics much less enjoyable than their other subjects (β_0_ =  − 22.4). This group of students reported a positive change in attitude (β_1_ =  + 5.2) following the Maker experience. Interestingly, while three clusters of students were also identified in the enjoyability SARs for science (Fig. 2), the trajectories for science are different to the trajectories for mathematics (Fig. 5). For science, the most notable changes in attitude were apparent in the group with initially neutral reports of enjoyability; for mathematics, it was the high and low enjoyability groups that show the most notable changes in attitude. The reasons for this are again unclear, but it again points to the need for more nuanced understandings of the impact that alternative pedagogies such as Maker activities can have.

As with science, Fig. 6 shows only small changes in students’ attitudes towards the relevance of mathematics following the Maker experience. Once again, the analysis identifies three clusters of students: a highly positive group (Cluster C, β_0_ = 61.8) of only two students, a moderately positive group (cluster A, β_0_ = 17.6) containing the majority of students, and a moderately negative group (cluster B, β_0_ =  − 13.8) containing the remaining quarter of students. Interestingly, while the changes are small, the attitude trajectories indicate a shift towards neutral attitudes. The negative group shows a small positive change (β_1_ =  + 1.4) while both positive groups show a small negative change in attitude (β_1A_ =  − 1.1, β_1C_ =  − 1.8). Of particular note however, is the apparent absence of a group of students holding neutral attitudes towards the relevance of mathematics. It seems that this class of Year 7 students holds attitudes towards the relevance of mathematics that are either more positive or more negative than their perceptions of the relevance of their school subjects as a whole, and that while short experiences like the visit to TICS can impact on students’ attitudes overall, they have little impact on their conceptions of relevance.

## Modes of making

As noted earlier, the findings we have reported above are part of an ongoing *educational design research* project being undertaken in partnership with the College. Educational design research, also known as design-based research, is a research framework noted for its commitment to developing theoretical insights and practical solutions simultaneously (McKenney & Reeves, 2012). Educational design research is iterative and responsively grounded. It seeks to explore rather than mute the complexities of real learning environments, meaning that projects typically change course in light of empirical data.

If the first half of this paper was an empirical report on a practical cycle of our project, then this second half of the paper offers a theoretical exploration intended to support future work. It is the thinking that goes into changing course. Notably, while we are reflecting on a project in Australia, the exploration is international—it is an extension of conversations between the Italian and Australian authors of the paper. Before we get into the theory though, let us pause to consider the different modes in which Making has emerged, and the challenges that each pose in light of the mixed responses we have reported above.

**Fig. 7:**
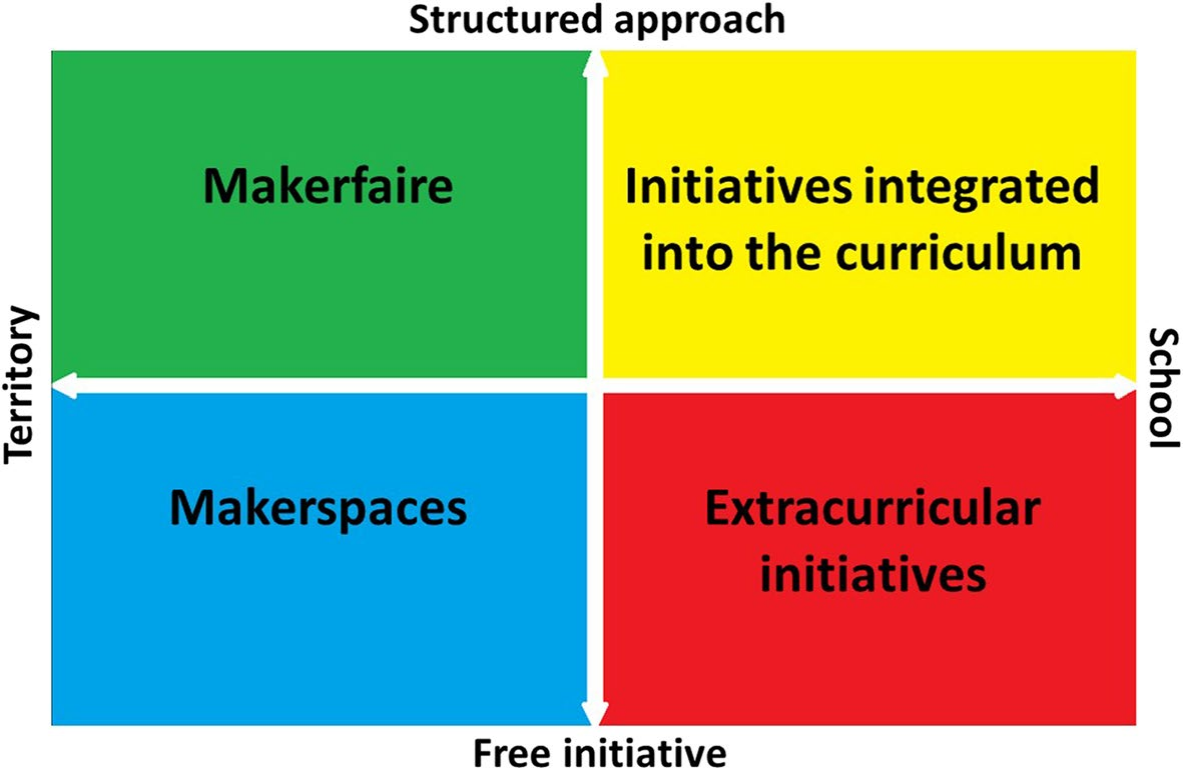
Modes of Making

In Fig. 7 we present these Making modes with respect to their level of teacher or student direction on the y-axis, and the level to which the activity is driven by the school or from beyond the school on the x-axis.

The approach chosen in the study reported in this paper sits in the lower left quadrant of Fig. 7. The TICS facility provided dedicated classroom spaces equipped with technologies and materials to house Maker activities, but the activities still looked a lot like “normal” school. They were heavily specified to encourage “success” and intended to drive learning outcomes connected to the regular curriculum and to allow formal assessment modes that are understood by teachers, students and parents.

There are clear advantages to this approach. Teachers can promote genuinely interdisciplinary approaches, for example (Karppinen et al., 2019). Further, it is not exclusionary. All students can be required to participate regardless of their perceived ability or their access. On the other hand, this approach presents the risk that over-structured activities may deliver qualitatively mediocre learning experiences which are disconnected from students’ experiences outside the school environment—a factor that may be driving the mixed student response we saw in this study.

One way that Making has sought to increase engagement by moving away from normal schooling is though the Maker Fair movement. Maker Fairs are typically seen as competitions conducted at a regional or national level. These structured projects are usually carried out by individuals or groups and have the great educational advantage of extended developmental time periods. Maker Fairs provide an excellent opportunity for students to engage with STEM in meaningful ways, but they are also more likely to be discriminatory as it is often only students with high academic achievement that are encouraged to be involved. Maker Fairs, however, could be opened to a wider group of participants if they can find diverse foci for diverse student engagement.

Many schools have also been open to giving students much greater freedom in their Making by opening up spaces like TICS to extra-curricular use, such as through a lunch time or after school club. Such clubs usually allow the student full freedom in experimenting with materials and technologies and in the creation of physical or digital artifacts (Jones, 2020). They can essentially be Maker Fairs without the competition. The assets developed in these programs are typically not valued—they are not formally assessed—but the students can be followed or mentored by expert Makers who take a more or less active role in supporting the students. Like Maker Fairs, though, a limitation of this approach is that it will often be chosen only by students who have a specific interest in scientific or technological disciplines. In order to make these activities more attractive for students at risk, who often avoid extra-school hours activities, the challenge that lays ahead for design and research is to define strategies to make them more motivating and inclusive. One pathway towards this may be the adoption of more culturally sensitive approaches that take into account the needs of diverse students.

Finally, activities located in the fourth, upper-right quadrant focus on approaches oriented towards cultural pluralism and involve students from the communities to which they belong. Makerspaces, also called FabLabs (Dreessen & Schepers, 2019; Walter-Herrmann & Büching, 2014), are born with this in mind and aim to cultivate and develop ideas that take on relevance within the community of learners and educators. These learning environments reflect the cultural peculiarities, identities and practices of that specific community. Grants are provided by third parties such as non-governmental organizations, non-profit associations and foundations, or by public entities such as museums and civic libraries to establish these centres in critical areas of cities or in the suburbs. These Makerspaces are free of both the constraints and requirements of formal education and of the need for formal assessment and evaluation of the products of learning. However, this lack of formality, may in turn lead to some disadvantaged students, who may not self-identify with the STEM disciplines, being unable or unwilling to engage with these activities.

## Discussion

A notable finding in this research is that the student response to the Maker activity was ranged. That is, the students with initially high attitudes tended to respond differently to students with initially low attitudes, and students with initially neutral attitudes responded differently again. In the study reported here we worked with a small cohort. We are not, therefore, suggesting that “high attitude” students will always respond to Maker activity as *this* group of high attitude students did. It is clear from this study, though, that a ranged response may be a likely outcome in other contexts, and that such a differentiated response needs to be considered in future iterations of this and other projects.

The reasons for this ranged response may be the simple idiosyncrasies of personal interest. The tendency for within-group similarities, however, makes this unlikely and suggests that there are some common drivers present. Clearly further empirical research is required, but the extant research literature also provides a sound basis for theoretical conjecture. There are logical mechanisms, for example, for disadvantage to be implicated in driving differentiated responses to Maker learning. We note, for example, that students from disadvantaged backgrounds are more likely than their wealthier peers to experience chronic and cumulative stressors that disrupt the self-regulation that is clearly important for success in Maker activities (Evans & Kim, 2013; O'Connor et al., 2019); and that there appears to be a relationship between disadvantage and emotional and behavioural problems that seem likely to impact on a positive engagement in the less routinised pedagogies of the Maker space (Flouri et al., 2014).

When considering learning in a complex system, though, our attention must not sit upon the students and their capacities alone. While effective cognitive and behavioural self-regulation are essential conditions for the open, collaborative and experimental nature of Maker learning, it is essential to note that these practices largely sit at odds with the traditional rigidity of the school curriculum and the structural and organizational constraints of schooling (Godhe et al., 2019). The desire for simple and clearly defined curriculum objectives in particular are not easily translated to Maker activities (Tan, 2018). The Maker movement asks not only for high levels of self-regulation, but also for teachers and learners to be able to traverse a culture gap that many will not have the socio-cultural resources to take on.

The literature skimmed in this paper suggests a theoretical way forward in light of the mixed responses our Maker activities have elicited. Instead of designing activities based on culturally dominant norms and asking why students at risk are not engaged, perhaps it is necessary to reverse the perspective and ask which activities are considered most significant to all students.

Maker activities designed with reference to fair, inclusive and non-competitive frameworks may have the potential to encourage even those who may not identify with STEM subjects in mainstream curriculum delivery. Martin et al. (2018), for example, take the students' practice repertoires or modalities of involvement in cultural activities as their main construct. This view would suggest a socio-cultural and constructivist perspective in which learning takes place in the context of prior knowledge of skills, symbolic systems and meanings. Students would take on the role of *active agents* being both designers of the spaces and objects of their learning (Bevan, 2017). They would be able to determine what can take on value in their projects and with whom to collaborate, leaving them free to incorporate their own repertoires of practice in the realization of projects.

## Conclusion

In the theoretical framework we have begun developing in this paper the concepts of agency, iteration and risk assumption take on particular relevance (Hsu et al., 2017). The agency that students should take on is based on constructionist approaches that are foundational for Maker movement and that provide the pedagogical and inclusive orientation often missing from neoliberal interpretations. The use of technologies such as 3D printers, Arduino boards, or laser cutters promotes what Vossoughi et al. (2016) define an epistemological pluralism emerging from new ways of exploring interdisciplinary topics. Mathematical concepts or physical phenomena, for example, can be explored through the construction of physical and digital objects, with a flexibility that allows each student in the role of scientist, artist or designer, to be able to express themselves driven by their own curiosity. At the same time, the activity does not leave the initiative entirely to the student but rather structures it in such a way that the support of the teacher or expert Maker acts as scaffolding for the development and testing of specific ideas.

The frustration resulting from having made a mistake is essential to activating the recursive feedback process that characterizes Maker activities and that leads to new solutions. By solving authentic problems, which refer to their daily life, students learn, develop new skills and build new meanings (Vuorikari et al., 2019). Within this process of creation, experimentation, debugging and recreation, iterative development takes on a crucial role in challenging the normative visions of knowledge production (Barton & Tan, 2018, 2019). Iteration however does not need to be limited to the construction of artifacts and for solving problems, it can help take into account the socio-cultural context of the students by using a critical ethnographic lens. This approach uses Maker movement to explore, legitimise and ultimately integrate the local knowledge and practices of those at risk into the decision-making process that drives change over iterations of design. The Maker environment can then be created in areas at risk of marginalization to involve the community and the students in the planning of activities and the management of the environments themselves.

The framework emerging from this study, if further developed, could provide a stronger theoretical foundation made of principled practice knowledge and heuristics for transforming Maker movement. Furthermore, it could guide the design and implementation of novel initiatives related to Maker movement, imprinting them on equity, inclusiveness and on the enhancement of the hidden talents of everybody, included the disadvantaged students.

## Data Availability

The data that support the findings of this study are available on request from the corresponding author. The data are not publicly available due to their containing information that could compromise the privacy of research participants.
